# Comparison of Visual Stimuli for Steady-State Visual Evoked Potential-Based Brain-Computer Interfaces in Virtual Reality Environment in terms of Classification Accuracy and Visual Comfort

**DOI:** 10.1155/2019/9680697

**Published:** 2019-07-01

**Authors:** Kang-min Choi, Seonghun Park, Chang-Hwan Im

**Affiliations:** Department of Biomedical Engineering, Hanyang University, 222 Wangsimni-ro, Seongdong-gu, Seoul 04763, Republic of Korea

## Abstract

Recent studies on brain-computer interfaces (BCIs) based on the steady-state visual evoked potential (SSVEP) have demonstrated their use to control objects or generate commands in virtual reality (VR) environments. However, most SSVEP-based BCI studies performed in VR environments have adopted visual stimuli that are typically used in conventional LCD environments without considering the differences in the rendering devices (head-mounted displays (HMDs) used in the VR environments). The proximity between the visual stimuli and the eyes in HMDs can readily cause eyestrain, degrading the overall performance of SSVEP-based BCIs. Therefore, in the present study, we have tested two different types of visual stimuli—pattern-reversal checkerboard stimulus (PRCS) and grow/shrink stimulus (GSS)—on young healthy participants wearing HMDs. Preliminary experiments were conducted to investigate the visual comfort of each participant during the presentation of the visual stimuli. In subsequent online avatar control experiments, we observed considerable differences in the classification accuracy of individual participants based on the type of visual stimuli used to elicit SSVEP. Interestingly, there was a close relationship between the subjective visual comfort score and the online performance of the SSVEP-based BCI: most participants showed better classification accuracy under visual stimulus they were more comfortable with. Our experimental results suggest the importance of an appropriate visual stimulus to enhance the overall performance of the SSVEP-based BCIs in VR environments. In addition, it is expected that the appropriate visual stimulus for a certain user might be readily selected by surveying the user's visual comfort for different visual stimuli, without the need for the actual BCI experiments.

## 1. Introduction

Electroencephalography (EEG) has been the most widely used neural signal for brain-computer interfaces (BCIs), whose main aim is to provide the paralyzed or disabled with new means of communication with the external environment [1]. Typical paradigms for EEG-based BCIs include motor imagery (MI), P300, and steady-state visual evoked potential (SSVEP) [2]. Among these, an SSVEP-based BCI paradigm has been widely employed because of its robustness to external noises and very little training requirement [3]. Owing to its advantages over the other paradigms and recent development of advanced analysis methods [4, 5], the SSVEP-based BCIs have been implemented for a variety of applications including assistive and rehabilitation tools for the disabled [6] and practical applications for the healthy, such as car navigation [7] and entertainment [8]. Furthermore, with the rapid advancements in the virtual reality (VR) technology, the SSVEP-based BCIs have been successfully applied to VR applications with hand-free control of the VR objects or speechless communications [9–11].

Although most VR devices currently employ head-mounted displays (HMDs), no previous SSVEP-based BCI study has considered the environmental differences between the VR-HMDs and conventional LCD monitors. Since the traditional SSVEP-based BCIs have used an LCD monitor as the rendering device to present visual stimuli for the past decades, a number of studies have already been conducted on the influence of the various parameters of this visual stimulus on the performance of the BCIs; these parameters include spatial frequency [12], temporal frequencies [13], colors [14], data recording channels [15], and time window sizes [16, 17]. On the contrary, the SSVEP-based BCIs implemented in VR environments have employed visual stimuli identical to those used in conventional LCD monitor environments, without any major modification. In other words, all SSVEP-based BCI studies performed in VR environments assumed that the presentation of visual stimuli on HMD is not significantly different from that on an LCD monitor. For example, MindBalance game [9], a 3D video game using SSVEP-based BCIs in VR environments, employed pattern-reversal checkerboard stimulus (PRCS) to elicit SSVEP response. A recently developed neuro-optical diagnostic tool using the VR headset [18] also employed the conventional PRCS. However, it is well known that an experiment in the VR environment is highly vulnerable to visual fatigue than that in the LCD environment; this is mainly due to the image distortion, or crosstalk, in the stereoscopic viewing [19] as well as the proximity between the source of illumination and the eyes [20].

In the present study, we have used two different types of visual stimuli—PRCS and grow/shrink stimulus (GSS)—both of which are known to effectively elicit SSVEP responses in the LCD monitor environment, on 14 participants wearing HMDs. The performance of the two representative visual stimuli was then investigated in terms of individual classification accuracy and subjective visual comfort scores. After the survey of the visual comfort of the participants in the preliminary offline experiments, the performance of SSVEP-based BCIs was investigated through online avatar control experiments in a VR environment.

## 2. Materials and Methods

### 2.1. Participants

Sixteen young, healthy people (10 males and 6 females, aged 20.5 ± 1.6 years) with normal or corrected-to-normal vision participated in our experiment. All participants were informed of the details of the experiments and had given their written consent. The data of two participants were excluded in further analyses: the first was excluded owing to the frequent blinking of the eyes during the presentation of the visual stimuli (eye blinks contaminated 14 out of the total 40 trials) and the second owing to the nonexistence of spectral peaks in the recorded EEG. The eye blinks were identified by visually inspecting vertical electrooculogram (EOG) recorded during the offline experiment. This so-called “BCI-illiteracy” is a well-known issue in EEG-based BCIs [21]. This experiment was approved by the institutional review board of Hanyang University, Republic of Korea (IRB HYI-14-167-11).

### 2.2. Visual Stimuli

**Figure 1:**
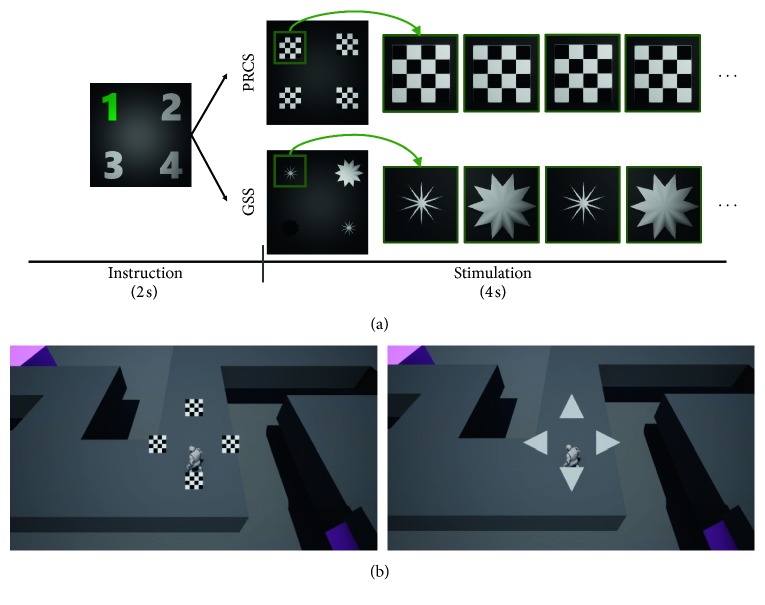
(a) Overall timeline for a single trial in offline experiments. In each trial, after a 2 s instruction period to inform participants of the location of a target visual stimulus, four visual stimuli, each of which was either PRCS or GSS, were presented for 4 s. (b) Screenshots of online experiments. The left picture was taken when the PRCS was employed, while the right picture was taken when the GSS was employed. The human avatar needed to move along a designated path (e.g., in the left direction in both figures).

Two different types of visual stimuli were employed to elicit SSVEP responses: a PRCS and a GSS. The PRCS is a traditional visual stimulus, which is used most frequently to elicit SSVEP responses in LCD monitor environments; this stimulus alternately presents two checkerboard patterns with 180° phase difference [7] (Figure 1(a)). The GSS is a new visual stimulus that changes both luminance and size to elicit SSVEP responses. This stimulus was based on previous studies, which reported that motional changes can also elicit periodic VEP responses (often referred to as steady-state motion visual evoked potential or SSMVEP) [22, 23] (Figure 1(a)). These stimuli were presented in a VR environment using an HMD of the HTC VIVE™ VR system (HTC Co., Ltd., Xindian District, New Taipei City, Taiwan). Both visual stimuli were modulated to elicit SSVEP responses corresponding to four frequencies, namely, 6, 7.5, 9, and 10 Hz. These frequencies were determined by considering the refresh rate of the rendering device (90 Hz), which is an integer multiple of each of the four target frequencies. In the offline experiments, the visual angle of the PRCS was fixed at 14°, while that of the GSS was varied between 8° and 16°. In the online experiments, the visual angle of the PRCS was reduced to 6° and that of GSS was varied between 4° and 8° in order to validate the feasibility and usability of the visual stimuli in a realistic VR environment in which large-sized stimuli cannot be generally employed. Note that according to previous reports, visual stimuli with visual angles greater than 3.8° would produce similar levels of SSVEP responses [24].

### 2.3. Experimental Paradigm

In the preliminary offline experiments, each stimulus type was presented in a randomly shuffled order to each participant. In each trial, four visual stimuli with different frequencies were presented for 4 s, as shown in Figure 1(a). The interstimulus interval (ISI) was set to 2 s, during which one of the numbers presented on the screen was colored green and flickered at 1 Hz to indicate the stimulus that the participant should focus on during the next stimulus interval. Each visual stimulus in each stimulus type appeared 20 times (five times for each frequency), and thus, the total number of trials was 40. The EEG signals were recorded; however, no immediate feedback was delivered to the participants during the experiment. At the end of the preliminary offline experiment, the participants were asked to subjectively rate their visual comfort with the two stimulus types on a scale ranging from 0 (very uncomfortable) to 10 (very comfortable).

In the online experiments, the participants who also participated in the preliminary offline experiments were asked to control a human full body avatar standing on a virtual road in a VR environment. The avatar could move in four directions: top, bottom, left, and right. Four visual stimuli with the frequencies used in the offline experiment were presented at the top, bottom, left, and right of the avatar to indicate the possible movement directions of the avatar (Figure 1(b)). Each participant was asked to sequentially move the avatar in a correct direction following the given path. A total of three different paths, each consisting of 20 movement steps, were created. For all 60 movement steps, the numbers of each directional step were counterbalanced. For each path, the same paradigm was repeated twice with either PRCS or GSS, when the presentation order of the visual stimuli was randomly determined for each participant. The avatar could move a step forward only when the classification result (direction) coincided with the correct direction of the path. Consequently, the minimum number of trials required to complete each session was 20, when the classification accuracy was 100%. Each trial lasted for 5 s, including 2 s for the presentation of the visual stimuli, 1 s for avatar's movement, and 2 s for ISI to give participants the time to shift their gaze for the next movement. A video clip showing the online experiment of a participant is attached to this manuscript as a Supplementary Movie, and its high resolution version can be found at YouTube™ (https://youtu.be/TC4QMPhW6y8).

### 2.4. Biosignal Acquisition and Preprocessing

The EEG data were recorded from seven electrodes (C_z_, PO_3_, PO_z_, PO_4_, O_1_, O_z_, and O_2_) using a commercial biosignal recording system (ActiveTwo, BioSemi, Amsterdam, and the Netherlands). In addition, a pair of electrodes was attached above and below the right eye to acquire the vertical EOG data. The sampling rate was set at 2,048 Hz. The recorded EEG data were re-referenced to C_z_ [4, 25] and then band-pass filtered at 6 and 50 Hz using a zero-phase Chebyshev type I infinite impulse response filter implemented in MATLAB (MathWorks, Inc., Natick, MA, USA). The program to analyze data in real time was developed using the FieldTrip toolbox [26].

### 2.5. Data Analysis and Statistical Analysis

For the classification of the SSVEP responses, we adopted a recently introduced algorithm called the extension of the multivariate synchronization index (EMSI) [5], which has exhibited outstanding performance compared to the conventional classification methods [27].

The Wilcoxon signed-rank test was employed for the statistical analysis because the classification accuracies with respect to the two visual stimulus types did not follow normal distribution as assessed by the Kolmogorov–Smirnov test.

## 3. Results

**Figure 2:**
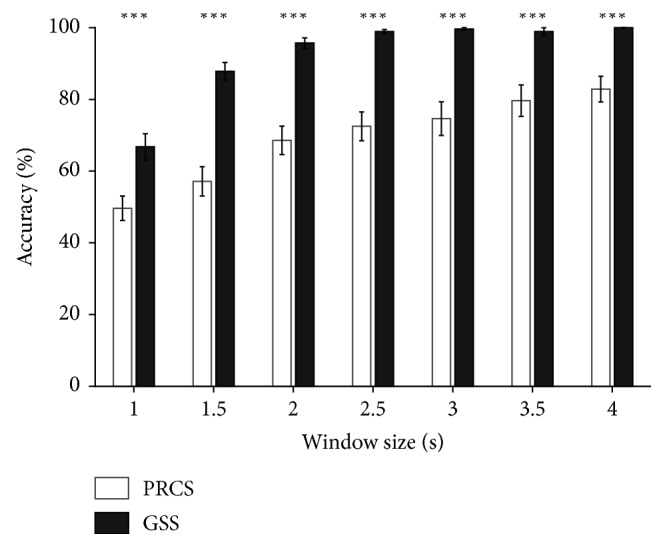
Comparison of offline experimental results between the PRCS and GSS in terms of the average classification accuracy across participants. The error bars indicate the standard errors. ^*∗∗∗*^ *p* < 0.005.

**Figure 3:**
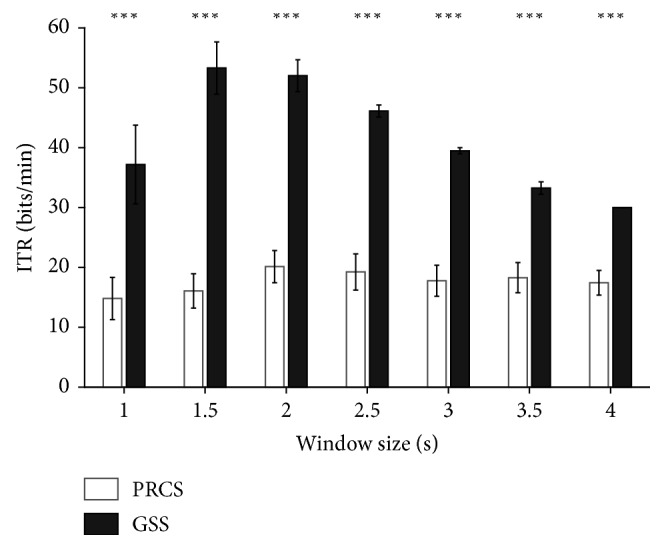
Comparison of offline experimental results between the PRCS and GSS in terms of the average ITR across participants. The error bars indicate the standard errors. ^*∗∗∗*^ *p* < 0.005.

In the offline experiment, the GSS outperformed the PRCS in both classification accuracy and information transfer rate (ITR) for all window sizes (Figures 2 and 3); ITR was calculated as follows:

(1) $$\begin{array}{c} \text{ITR}=\frac{\left(\log_{2}\;N+P\;\log_{2}\;P+\left(1-P\right)\log_{2}\left(1-P\right)/\left(N-1\right)\right)}{C}, \end{array}$$

where *N* denotes the number of stimuli, *P* denotes the classification accuracy ranging from 0 to 1, and *C* denotes the time needed to classify a single trial [28]. Statistical analysis using the Wilcoxon signed-rank test also showed statistically significant difference in the performance of the GSS and PRCS (Bonferroni-corrected *p* < 0.005 for both classification accuracy and ITR for all window sizes). Although a window size of 1.5 s showed the highest ITR (Figure 3), 2 s epochs were used for the classification in the online experiments. This was because the difference between the ITRs for the 1.5 s and 2 s epochs was not big, but the improvement in the classification accuracy was relatively distinct for the 2 s epoch compared with the 1.5 s epoch.

Table 1 shows the classification accuracy of each participant in the online experiment. Unlike the preliminary offline experiment, no statistical significance was observed between the classification accuracies for the PRCS and GSS (*p*=0.424; Wilcoxon signed-rank test) in the online experiment although the average classification accuracy for the GSS was higher than that for the PRCS. The possible reasons for the difference between the two cases, i.e., the offline and online experiments, will be discussed in Discussion.

For further analyses, all participants were divided into three groups based on the subjective visual comfort ratings for the two visual stimulus types that were obtained right after the preliminary offline experiment. The participants who were more comfortable with the PRCS were categorized as Group 1, and those who were more comfortable with the GSS were categorized as Group 2. The participants who rated both stimuli equally were categorized as Group 3 and excluded from further analyses. Interestingly, all three participants (i.e., P6, P8, and P10) in Group 1 exhibited higher classification accuracies for the PRCS than for the GSS, while most participants in Group 2, with the exception of only one participant (i.e., P5), exhibited higher or equivalent classification accuracies for the GSS than for the PRCS. These results suggest that the performance of the SSVEP-based BCIs in VR environments might be potentially improved by selecting the best stimulus type for each individual, which would be readily chosen by inspecting the individual's subjective visual comfort for different visual stimuli types.

## 4. Discussion

The performances of the reactive BCI systems are highly dependent on the types of stimuli used to elicit specific EEG responses. Although a series of studies has been performed to find an optimal visual stimulus for the conventional SSVEP-based BCIs in the LCD monitor environment, no study has yet been reported on the influence of visual stimuli on the performance of the SSVEP-based BCIs in VR-HMD environments. We hypothesized that the PRCS, which are widely used in the SSVEP-based BCIs, might not be the optimal visual stimulus in a VR-HMD environment because the images displayed on the HMDs are closer to the eyes than those on the LCD monitors, and thus, the PRCS might be too intense for the eyes. Therefore, in this study, we tested another type of visual stimulus called the GSS that changes both size and luminance in VR environments and compared the BCI performances with the PRCS.

In the offline experimental results, the GSS outperformed the PRCS in terms of classification accuracy; however, the difference in the performance was considerably reduced in the online experiments. This phenomenon is thought to originate from several factors: first, the spatial frequency of the PRCS in the offline experiment was different from that in the online experiment. The spatial frequency changed from 0.25 cycle/deg in the offline experiment to 0.5 cycle/deg in the online experiment. According to a previous report [12], spatial frequency of PRCS has close relationship with the performance of SSVEP-based BCIs. The second reason might be the difference in the background; for instance, in the offline experiment, a monotonous dark grey background was used, while in the online experiment, a relatively complicated background with many distractors was employed (Figure 1(b)). This complicated background might have distracted the elicitation of the SSMVEP because the border of the GSS sometimes becomes obscure owing to the background images. On the contrary, the PRCS would be less affected by the background because this stimulus maintains its size during the presentation.

Our online experiments demonstrated that the SSVEP-based BCI with a visual stimulus that was more comfortable for the user generally outperformed that with the other stimulus in VR environment. This finding is not in line with previous reports showing that a visual stimulus evoking stronger SSVEP responses induced the severer visual fatigue [29–31] when an LCD monitor was used for presenting visual stimuli. However, there are also some evidences showing that the relationship between visual comfort and BCI performance is dependent upon the stimulation rendering device (e.g., light emitting diodes: LEDs) or stimulus types (e.g., SSMVEP) [32, 33]. Our results also suggest that a user's optimal visual stimulus in VR environments might be readily determined by rating the subjective visual comfort of the user even before the main BCI experiment. This strategy might considerably alleviate the necessity of a series of offline BCI experiments to determine an optimal visual stimulus for the user in the VR environment.

In the offline experiment, four participants rated the same visual comfort score for both PRCS and GSS. Interestingly, they commonly achieved better classification accuracies in the GSS than in the PRCS. Although the limited sample size makes it hard to generalize, selecting GSS rather than PRCS might yield better classification accuracies in cases when there is no difference in the subjective visual comfort ratings. However, further investigations are required to formulate a more generalized rule for selecting the optimal visual stimulus for the SSVEP-based BCIs in VR environments. In addition, in the present study, we tested only two types of visual stimuli; however, more types of visual stimuli need to be developed and tested in VR environments in future studies.

## 5. Conclusions

To the best of our knowledge, this is the first study that has compared different types of visual stimuli for the SSVEP-based BCIs in VR environments. Our study demonstrated that selection of an optimal visual stimulus for an individual could improve the overall performance of the SSVEP-based BCIs and reduce visual fatigue in VR environment. A close association between the performance of the SSVEP-based BCIs and subjective visual comfort was observed, suggesting that the selection of an appropriate visual stimulus via a simple pre-experimental inspection of the individual's preference toward the visual stimuli might help to enhance the performance of the SSVEP-based BCIs in VR environments.

## Figures and Tables

**Table 1:** Comparison of online classification accuracies between the PRCS and GSS.

Group	Participant	PRCS accuracy (%)	GSS accuracy (%)
Group 1	P6	84.5	58.8
	P8	92.3	85.7
	P10	98.4	92.3

Group 2	P2	100	100
	P3	66.7	98.4
	P5	96.8	76.9
	P7	100	98.4
	P11	89.6	98.4
	P12	80.0	80.0
	P13	84.5	95.2

Group 3	P1	70.6	89.6
	P4	84.5	90.9
	P9	75.9	92.3
	P14	83.3	96.8

Average ± std.		86.2 ± 10.7	89.6 ± 11.3

Group 1 includes participants who rated PRCS as more comfortable to their eyes than GSS. Group 2 includes participants who rated GSS as more comfortable than PRCS. The remaining participants who gave the same score to both stimuli are categorized as Group 3.

## Data Availability

The data used to support the findings of this study can be made available form the corresponding author upon request.

## Supplementary Materials

Supplementary Materials A movie clip showing the online experiment is attached to this manuscript. Two consecutive trials for each type of stimuli were recorded as a video clip to illustrate the brief paradigm of this experiment: one for the case of the classification result being correct, while the other for that being incorrect. As mentioned in Materials and Methods, the avatar moved only in cases when the classification result coincided with the direction of the path.

## References

[1] Wolpaw J. R., Birbaumer N., McFarland D. J., Pfurtscheller G., Vaughan T. M. (2002). Brain-computer interfaces for communication and control. *Clinical Neurophysiology*.

[2] Yin E., Zeyl T., Saab R., Chau T., Hu D., Zhou Z. (2015). A hybrid brain-computer interface based on the fusion of P300 and SSVEP scores. *IEEE Transactions on Neural Systems and Rehabilitation Engineering*.

[3] Bin G., Gao X., Yan Z., Hong B., Gao S. (2009). An online multi-channel SSVEP-based brain–computer interface using a canonical correlation analysis method. *Journal of Neural Engineering*.

[4] Zhang Y., Yin E., Li F. (2018). Two-stage frequency recognition method based on correlated component analysis for SSVEP-based BCI. *IEEE Transactions on Neural Systems and Rehabilitation Engineering*.

[5] Zhang Y., Guo D., Yao D., Xu P. (2017). The extension of multivariate synchronization index method for SSVEP-based BCI. *Neurocomputing*.

[6] Lesenfants D., Habbal D., Lugo Z. (2014). An independent SSVEP-based brain–computer interface in locked-in syndrome. *Journal of Neural Engineering*.

[7] Martinez P., Bakardjian H., Cichocki A. (2007). Fully online multicommand brain-computer interface with visual neurofeedback using SSVEP paradigm. *Computational Intelligence and Neuroscience*.

[8] Van Vliet M., Robben A., Chumerin N. Designing a brain-computer interface controlled video-game using consumer grade EEG hardware.

[9] Lalor E. C., Kelly S. P., Finucane C. (2005). Steady-state VEP-based brain-computer interface control in an immersive 3D gaming environment. *EURASIP Journal on Advances in Signal Processing*.

[10] Coogan C. G., He B. (2018). Brain-computer interface control in a virtual reality environment and applications for the internet of things. *IEEE Access*.

[11] Faller J., Allison B. Z., Brunner C. (2017). A feasibility study on SSVEP-based interaction with motivating and immersive virtual and augmented reality. https://arxiv.org/abs/1701.03981.

[12] Waytowich N. R., Yamani Y., Krusienski D. J. (2017). Optimization of checkerboard spatial frequencies for steady-state visual evoked potential brain-computer interfaces. *IEEE Transactions on Neural Systems and Rehabilitation Engineering*.

[13] Waytowich N. R., Krusienski D. J. Novel characterization of the steady-state visual evoked potential spectrum of EEG.

[14] Gerloff M., Schilling M. (2012). Subject response variability in terms of colour and frequency of capacitive SSVEP measurements. *Biomedical Engineering/Biomedizinische Technik*.

[15] Hwang H.-J., Lim J.-H., Jung Y.-J., Choi H., Lee S. W., Im C.-H. (2012). Development of an SSVEP-based BCI spelling system adopting a QWERTY-style LED keyboard. *Journal of Neuroscience Methods*.

[16] Yin E., Zhou Z., Jiang J., Yu Y., Hu D. (2015). A dynamically optimized SSVEP brain-computer interface (BCI) speller. *IEEE Transactions on Biomedical Engineering*.

[17] Jiang J., Yin E., Wang C., Xu M., Ming D. (2018). Incorporation of dynamic stopping strategy into the high-speed SSVEP-based BCIs. *Journal of Neural Engineering*.

[18] Versek C., Rissmiller A., Tran A. (2019). Portable system for neuro-optical diagnostics using virtual reality display. *Military Medicine*.

[19] Bando T., Iijima A., Yano S. (2012). Visual fatigue caused by stereoscopic images and the search for the requirement to prevent them: a review. *Displays*.

[20] Guo J., Weng D., Duh H. B.-L., Liu Y., Wang Y. Effects of using HMDs on visual fatigue in virtual environments.

[21] Allison B., Luth T., Valbuena D., Teymourian A., Volosyak I., Graser A. (2010). BCI demographics: how many (and what kinds of) people can use an SSVEP BCI?. *IEEE Transactions on Neural Systems and Rehabilitation Engineering*.

[22] Xie J., Xu G., Wang J., Zhang F., Zhang Y. (2012). Steady-state motion visual evoked potentials produced by oscillating Newton’s rings: implications for brain-computer interfaces. *PLoS One*.

[23] Xie J., Xu G., Wang J. (2016). Effects of mental load and fatigue on steady-state evoked potential based brain computer interface tasks: a comparison of periodic flickering and motion-reversal based visual attention. *PLoS One*.

[24] Ng K. B., Bradley A. P., Cunnington R. (2012). Stimulus specificity of a steady-state visual-evoked potential-based brain–computer interface. *Journal of Neural Engineering*.

[25] Lai S. M., Zhang Z., Hung Y. S., Niu Z., Chang C. (2011). A chromatic transient visual evoked potential based encoding/decoding approach for brain-computer interface. *IEEE Journal on Emerging and Selected Topics in Circuits and Systems*.

[26] Oostenveld R., Fries P., Maris E., Schoffelen J.-M. (2011). FieldTrip: open source software for advanced analysis of MEG, EEG, and invasive electrophysiological data. *Computational Intelligence and Neuroscience*.

[27] Zhang Y., Xu P., Cheng K., Yao D. (2014). Multivariate synchronization index for frequency recognition of SSVEP-based brain-computer interface. *Journal of Neuroscience Methods*.

[28] Wolpaw J. R., Ramoser H., McFarland D. J., Pfurtscheller G. (1998). EEG-based communication: improved accuracy by response verification. *IEEE Transactions on Rehabilitation Engineering*.

[29] Duszyk A., Bierzyńska M., Radzikowska Z. (2014). Towards an optimization of stimulus parameters for brain-computer interfaces based on steady state visual evoked potentials. *PLoS One*.

[30] Dreyer A. M., Herrmann C. S., Rieger J. W. (2017). Tradeoff between user experience and BCI classification accuracy with frequency modulated steady-state visual evoked potentials. *Frontiers in Human Neuroscience*.

[31] Sakurada T., Kawase T., Komatsu T., Kansaku K. (2015). Use of high-frequency visual stimuli above the critical flicker frequency in a SSVEP-based BMI. *Clinical Neurophysiology*.

[32] Mouli S., Palaniappan R. (2017). Toward a reliable PWM-based light-emitting diode visual stimulus for improved SSVEP response with minimal visual fatigue. *Journal of Engineering*.

[33] Yan W., Xu G., Li M. (2017). Steady-state motion visual evoked potential (SSMVEP) based on equal luminance colored enhancement. *PLoS One*.

